# The Connectome Mapper: An Open-Source Processing Pipeline to Map Connectomes with MRI

**DOI:** 10.1371/journal.pone.0048121

**Published:** 2012-12-18

**Authors:** Alessandro Daducci, Stephan Gerhard, Alessandra Griffa, Alia Lemkaddem, Leila Cammoun, Xavier Gigandet, Reto Meuli, Patric Hagmann, Jean-Philippe Thiran

**Affiliations:** 1 Signal Processing Laboratory, Ecole Polytechnique Fédérale de Lausanne, Lausanne, Switzerland; 2 Department of Radiology, Lausanne University Hospital, and University of Lausanne, Lausanne, Switzerland; UCSF, United States of America

## Abstract

Researchers working in the field of global connectivity analysis using diffusion magnetic resonance imaging (MRI) can count on a wide selection of software packages for processing their data, with methods ranging from the reconstruction of the local intra-voxel axonal structure to the estimation of the trajectories of the underlying fibre tracts. However, each package is generally task-specific and uses its own conventions and file formats. In this article we present the Connectome Mapper, a software pipeline aimed at helping researchers through the tedious process of organising, processing and analysing diffusion MRI data to perform global brain connectivity analyses. Our pipeline is written in Python and is freely available as open-source at www.cmtk.org.

## Introduction

Since its advent, magnetic resonance imaging (MRI) has revolutionised the research in fundamental neuroscience. MRI is a non-irradiating and non-invasive imaging technique offering several modalities for studying the human brain from different angles, opening new perspectives previously unconceivable for studying the brain. Diffusion (dMRI) and functional (fMRI) magnetic resonance imaging are two well-established modalities providing powerful and complementary ways to investigate how different areas of the brain are interconnected and interact. In particular, dMRI exploits the thermal random motion of water molecules in biological tissues for mapping the local axonal structure at each imaging voxel [1], [2]. By using this information, fibre-tracking algorithms (also known as tractography) estimate trajectories capturing coherent orientations of maximal diffusion that are likely to represent real white matter fibre tracts linking together distinct grey matter areas of the brain [3], [4]. A comprehensive map of neural connections of the brain is called “*connectome*” [5], [6]. The connectome can be studied and described at different scales. At the macroscopic scale, the connectome can be seen as a *network*, where each *vertex* represents a well-defined cortical or sub-cortical structures and the *edges* quantify the structural white matter connectivity as measured with tractography. A connectome is usually represented by means of the *adjacency matrix* of the corresponding graph, also known as *connectivity matrix*, which is a square matrix summarising the connectivity for each pair of vertices. In the case when this matrix is estimated from dMRI data we speak about *structural connectivity*. On the other hand, the term *functional connectivity* is adopted if fMRI data is used instead [7], [8]. In this paper, we focus on structural connectivity and present a novel software for mapping connectomes.

**Figure 1:**
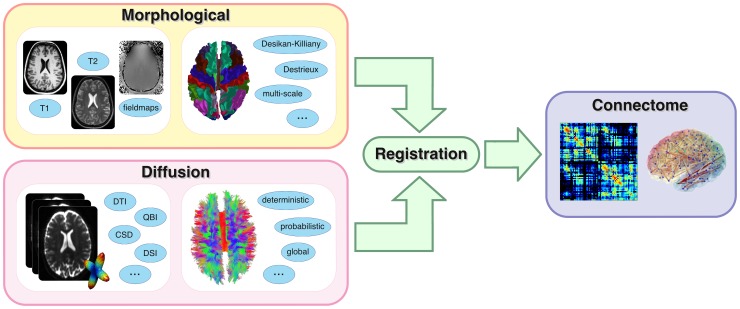
Basic workflow to create a connectome. Morphological and diffusion MRI images are processed as separate streams. Several possibilities are available in each stage. The final connectome is obtained by registering and merging the data coming from the two streams.

Schematically, three steps are necessary to compute a connectome from diffusion MRI data, as illustrated in figure 1. First, a morphological high-resolution T1-weighted image is used to segment the brain and identify different grey matter structures, such as the deep grey nuclei and cortical gyri, for obtaining the nodes of the network. Then, the macroscopic pathways of the underlying neuronal fibres (i.e. tractograms) need to be estimated from the diffusion data by means of tractography. Finally, the connectivity matrix is obtained by registering the two image spaces (i.e. morphological and diffusion) and then intersecting the estimated fibre trajectories with each pair of segmented cortical and sub-cortical regions.

At the practical level, however, each stage consists of several operations. There exist a vast choice of freely available software packages to solve each individual task, with tools for reconstructing the diffusion information in each voxel, performing fibre-tracking, segmenting the grey matter, registering images etc. However, most of these packages focus on a very specific task and use their own conventions, file formats and programming languages. Usually, researchers have to write custom scripts to combine these packages together to account for data format conversion and fulfil the requirements of each specific tool. This task can be rather daunting and, more importantly, does not favour data sharing. All these problems raised the need for standardisation and our software aims at addressing several of these issues.

To our knowledge, only very few frameworks exist for combining all these dedicated tools together in a sort of user-friendly processing pipeline. Nipype [9] and the Loni Pipeline [10] are two interesting frameworks developed for building custom processing workflows with little effort by selecting and combining modules from a wide range of well known neuroimaging software packages such as Fsl (www.fmrib.ox.ac.uk/fsl), Freesurfer (surfer.nmr.mgh.harvard.edu), Diffusion Toolkit (www.trackvis.org/dtk) etc. However, the primary goal of these tools is to quickly create custom processing workflows fulfilling specific needs, but they do not specifically address the creation of connectomes in a common and standardised way, thus favouring data sharing. Recently, Gray et al. [11] released a software pipeline based on Java Image Science Toolkit (JIST) which is very close to our tool. The main drawback of their approach is that it considers only Diffusion Tensor Imaging (DTI) data and other diffusion modalities such as Q-Ball Imaging (QBI) or Diffusion Spectrum Imaging (DSI) are not taken into consideration (see [12] for a good review on these modalities).

In this article we present the Connectome Mapper (CMP), a novel software tool whose main goal is to guide and help researchers through all the steps needed to compute connectomes. CMP simplifies the organisation, processing and statistical analysis of the data. It works transparently with some of the most used acquisition schemes (DTI, QBI and DSI) and its modular structure makes it easy to customise it for specific needs. Our software is developed in Python and it is designed to be fully compatible with many state-of-the-art software packages in this field.

The manuscript is organised as follows. In the next section we introduce the architecture of the Connectome Mapper and describe its main processing stages. Advantages and drawbacks of CMP are highlighted and discussed in the “Results and Discussion” section, where we also report clinical studies in which our software was successfully employed. We conclude this paper with some possible future directions we envision for the Connectome Mapper.

## Methods

**Figure 2:**
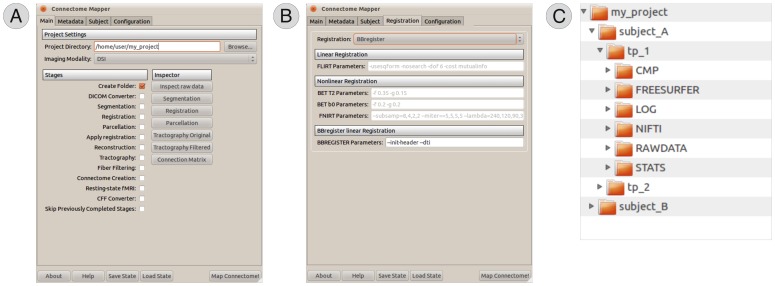
Graphical user interface (GUI) of the Connectome Mapper. The GUI controls the proper execution of the whole processing workflow (A) and helps the user in setting all the parameters required at each step (B). Metadata associated with the files being processed can also be entered, and CMP organises the data accordingly in a hierarchical structure (C) in order to simplify the management of big amount of data.

The Connectome Mapper implements a full processing pipeline for creating multi-variate and multi-resolution connectomes with dMRI data. The CMP has a modular structure composed of *processing stages*, each implementing a specific task of the workflow, and a *graphical user interface* (GUI) which supports the control and proper execution of these stages (figure 2A) and helps the user in the configuration of all the parameters required at each step (figure 2B). Metadata associated with the data being processed can also be entered (e.g. project, subject name etc), and all the files are organised accordingly in a hierarchical structure (figure 2C). All the necessary conversions among file formats are done under-the-hood completely transparent to the user. Where possible, CMP makes use of the native file format conversion tools bundled with every package, which is most often the case. Ad-hoc converters have been explicitly developed and included in CMP for any other proprietary file format.

**Figure 3:**
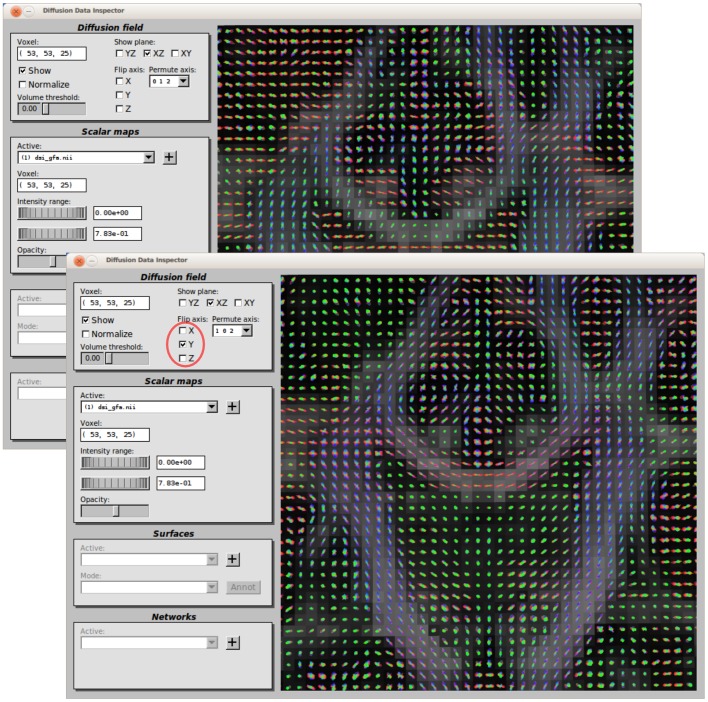
Data inspector. Sometimes a flipping/swapping can be present in the data due to incorrect information stored in the image's header (top). CMP allows to interactively explore the data and easily fix the problem (bottom).

After each step, the GUI offers the possibility to inspect the outputs generated and to repeat a specific step if the results are found to be flawed or not satisfactory. The user can tune any control parameter provided by the processing step, directly modify the intermediate data (e.g. manually correcting a mask) and then resume the processing at any time. In general, the outputs are inspected using native viewers bundled with each software package included in the workflow as the final user might be already very familiar with these tools. For instance, all MRI volume data can be inspected with the FslView viewer shipped with Fsl, whereas Trackvis is used to visualize the fibre bundles estimated with tractography. In addition, an internal viewer is included in CMP which provides additional features and complementary ways to inspect the outputs. This viewer (figure 3) allows to visualize in the same 3D space: (i) the diffusion data, in terms of glyphs representing the diffusion profiles in each voxel and identified maxima, (ii) additional MRI volume datasets, (iii) 3D surface models, like for instance the white-gray matter boundary extracted with Freesurfer and (iv) the final structural network estimated with CMP itself. Most of the figures presented in this manuscript have been produced using this viewer.

### Implementation

The Connectome Mapper is part of the Connectome Mapping Toolkit (CMTK), a complete software suite to map, visualise and analyse connectomes. It is implemented with the *Python programming language* and is released as open-source under BSD license. Documentation and source code are available at the following URL: www.cmtk.org. We chose Python since it is a free, open-source and cross-platform programming language which is rapidly becoming the language of choice for scientific computing [13]. In particular, CMP heavily relies on the Enthought Tool Suite (www.enthought.com), which is a comprehensive collection of open-source Python components providing all the necessary graphics and scientific libraries needed to develop the backbone and the graphical user interface of CMP.

We used Nibabel from the Neuroimaging in Python project (www.nipy.org) for I/O of neuroimaging file formats. For the GUI we used the Google Protocol Buffers and their Python bindings to define the interfaces of the processing stages; this ensures that the input files to each stage exist and that each stage produces all the required output files. CMP can also be scripted, that is, a configuration file can be created from the GUI in order to subsequently run the pipeline on a collection of datasets without the need to use the GUI. This is very important in order to guarantee a consistent and homogeneous processing across subjects when dealing with a large amount of data. Moreover, to reduce the total computation time, a group of subjects might be processed in parallel on several workstations or even on a computer cluster as separate jobs. However, CMP does not natively support any cluster management or job scheduling strategy and this operation is left to user.

### Main processing stages

Every processing stage uses a mixture of state-of-the-art neuroimaging tools, such as Fsl, Freesurfer and Diffusion Toolkit, and in-house developed scripts. Thanks to the modular nature of CMP, each stage can be easily customised to suit specific needs and new processing stages can be added with relatively little effort to account for additional algorithms. In the following, the main processing stages of CMP are described in detail.

#### Input data

The minimum pre-requisites to start the processing workflow are: (i) one diffusion acquisition and, (ii) one high-resolution morphological T1-weighted volume. Both DICOM (including mosaic images) and NIFTI image formats are supported; for convenience, all the processed data are internally converted to NIFTI. Regarding the diffusion images, the Connectome Mapper currently accepts a wide range of different acquisition schemes and provides a common way to seamlessly create connectomes independently of the acquisition scheme adopted. Project- and subject-related information can also be entered (figure 2). A tree folder structure is then created to hierarchically organise the input raw data and all the outputs generated at each processing stage.

The Connectome Mapper has been mainly tested with SIEMENS and GE data without encountering any particular problem. However, one of the most annoying practical issues when processing dMRI data is the presence, from time to time, of arbitrary flipping/swapping of some components of the diffusion gradient directions, as shown at the top of figure 3. They originate from incorrect information stored in the header of the images and result in wrong reconstructions normally difficult to debug. To address this issue, CMP offers the possibility to the user to manually specify the appropriate acquisition scheme as a text file (perhaps provided by the vendor). Moreover, an ad-hoc inspector window is provided for helping the user to figure out what went wrong and fix the problem (figure 3,bottom).

#### Whole brain tissue segmentation

The first processing step is the segmentation of the brain in white matter, grey matter (cortical and sub-cortical structures) and CSF starting from the high-resolution T1-weighted image. The extracted labels will serve later on as nodes of the connectome. By default CMP uses Freesurfer for this task, but additional or custom atlases can be easily added without breaking the workflow. Freesurfer reconstructs the folding structure of the cortex (sulci and gyri) very accurately, and provides an automatic labelling of the cortical and sub-cortical structures based on two different anatomical atlases (Desikan-Killiany and Destrieux).

**Figure 4:**
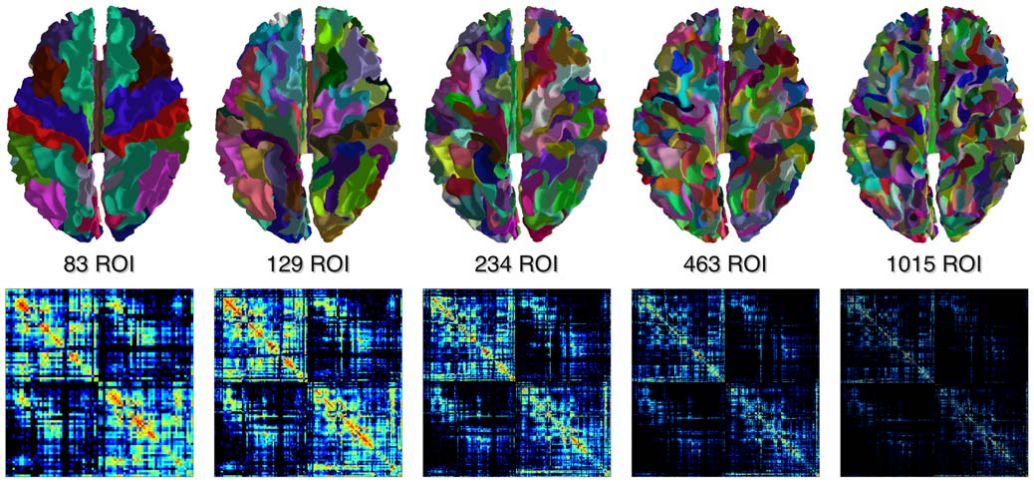
Multi-scale connectomes. The five multi-scale atlases derived from the Desikan-Killiany's anatomical atlas implemented in Freesurfer, and the corresponding connectivity matrices.

Starting from the Desikan-Killiany anatomical atlas and following the procedure described in [14], the cortical surface is further subdivided into parcels through a two-phase partitioning heuristic to create a *multi-scale parcellation* of the cerebral cortex. A total of five different subject-specific atlases were obtained by successive grouping of neighbouring regions at the next higher resolution (figure 4). At the smallest scale, the cortical parcels have approximately equal surface of 1.5 cm^2^. At the end of the process, each of the five atlases comprises, respectively, a total of 1015, 463, 234, 129 and 83 labels. The labels account for all the cortical structures, as well as the deep-grey nuclei and the brainstem. To our knowledge, the implementation included in our software represents the only freely-available approach to estimate multi-scales connectomes.

With such a parcellation it is then possible to generate whole brain normalised connectivity matrices at several scales (figure 4) and thus study the human connectome at different levels. In fact, the number of nodes has been shown to be an important feature for increasing the sensitivity in connectivity-based group studies on both structural and functional brain networks [15], [16].

#### Registration to diffusion space

**Figure 5:**
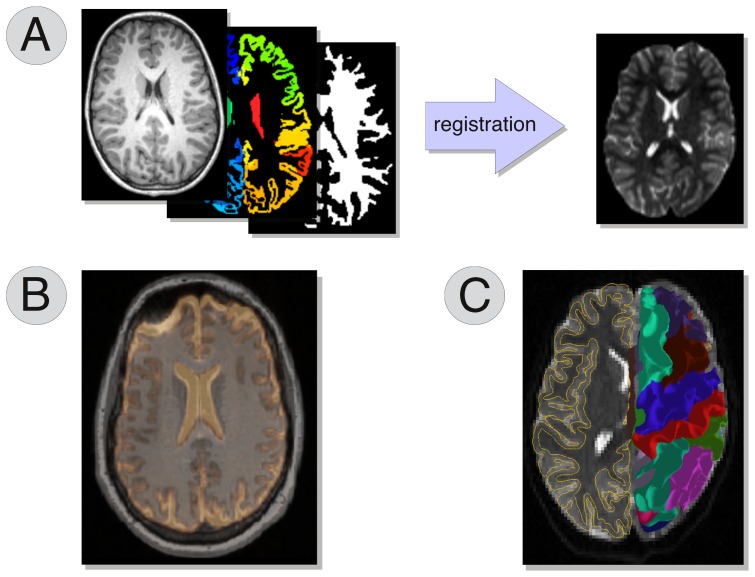
Registration between morphological and diffusion images. The reference space in CMP is the one of the diffusion images. The tissue masks extracted during the segmentation step, then, have to be registered to the diffusion space (A). The quality of the registrations can be inspected by overlaying on the b0 either the T1-weighted volume (B) or the geometric models of the cortex estimated with Freesurfer (C).

In the Connectome Mapper we use the diffusion acquisition as the reference space. Thus, the tissue masks created during the segmentation step have to be registered to the (eventually resampled, see next section) b0 volume, i.e. the volume acquired in absence of diffusion sensitising gradients, as shown in figure 5A.

Currently, three different registration options are available in the CMP. The simplest approach consists in linearly registering the T1-weighted image to the b0 volume. The affine transformation is estimated with the *intensity-based linear registration* tool available in Fsl, i.e. Flirt, which is fast and optimised to give good results in most cases. If needed, the GUI offers the possibility to tune all the parameters of the algorithm to adapt to the specific case, e.g. degrees of freedom, similarity function etc, and to check the quality of the registration after each run (figures 5B and 5C).

The second option is only available if Freesurfer has been used for the brain segmentation. In this case the registration can be improved using the *boundary-based registration* approach included in Freesurfer called bbregister. This method exploits the high-resolution geometric models of the cortex previously estimated and maximises the intensity gradient across different tissue boundaries. Although bbregister is based on linear transformations, it usually results in more robust registrations than the intensity-based method.

However, as diffusion images are normally affected by geometric distortions due to the EPI read-out [17], all the linear registration approaches are known to be sub-optimal. In fact, to correct for these non-linear distortions an additional scan, called *field-maps*, is required. CMP has a basic support for these field-maps, including a pre-processing step which relies on the Fugue tool of Fsl. In case the field-maps were not acquired, however, the linear registration can still be improved if a T2-weighted non-EPI acquisition is available (this is the case for most clinical protocols). In fact, such T2-weighted non-EPI images have much less distortions than the b0 volume and, importantly, share the same contrast with it. This property can be exploited to implicitly compensate for the geometric distortions by using the *non-linear registration*-based correction approach described in [18]. First, the T1-weighted volume is initially aligned with the T2-weighted non-EPI image with Flirt using 6 degrees of freedom. Subsequently, the T2-weighted volume is non-linearly registered to the b0 volume using Fnirt, the non-linear registration tool available in Fsl. Fnirt models the displacement field as sum of cubic splines and thus allows for more complex and accurate deformations between the images, in this way accounting also for the non-linear distortions. Finally, the two transformations are concatenated and used to register the T1-weighted volume as well as the previously estimated tissue masks to the diffusion space.

#### Intra-voxel reconstruction of diffusion information

CMP relies on Diffusion Toolkit to reconstruct the intra-voxel configuration of fibre compartments, and thus it natively supports the most popular acquisition schemes, ranging from the standard Diffusion Tensor Imaging (DTI) to more complex modalities like Q-Ball (QBI) and Diffusion Spectrum (DSI) Imaging. However, as already mentioned, the modular structure of CMP makes it straightforward to account for additional acquisition schemes by incorporating the corresponding reconstruction methods into the workflow. At the time of writing, additional reconstruction packages such as Mrtrix (www.brain.org.au/software/mrtrix) and Camino (cmic.cs.ucl.ac.uk/camino) are in the process of being included.

Raw diffusion MRI images can be up-sampled to any given resolution before reconstruction. By default, data is resampled to 2 mm isotropic voxel size using trilinear interpolation, but the user has the freedom to specify the desired resolution or to leave the data unchanged.

**Figure 6:**
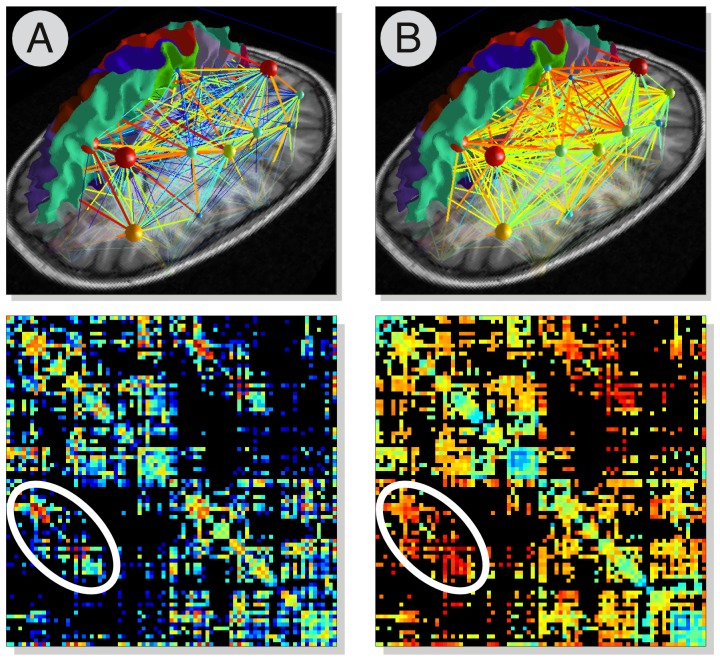
Multi-variate connectomes. Example of weighted connectomes computed using different measures for quantifying the connectivity strength between each pair of regions. In (A) the weight of each edge is proportional to the *number of connecting fibres* (logarithmic scale), while in (B) the *average GFA along the bundles* was used instead. The inter-hemispheric connections are highlighted here in white: although they do not differ much in number from the rest of the brain, they clearly manifest a higher GFA as they pass through the *Corpus Callosum*.

Depending on the scheme adopted, several quantities characterising the local diffusion process can be computed, such as the Apparent Diffusion Coefficient (ADC) and the Fractional Anisotropy (FA) of the diffusion tensor [2], [19], the Orientation Distribution Function (ODF) and its associated shape descriptors (Generalised Fractional Anisotropy, Skewness and Kurtosis [20]), or the full diffusion Ensemble Average Propagator (EAP) and its Zero-displacement Probability measure [21]. Later on, these quantities can be used for calculating the edges of the final brain network and creating a *weighted connectome*, as shown in figure 6.

#### Fibre-tracking

By default, whole-brain tractography is performed by means of a deterministic *streamline algorithm* as described in [22], [23], which accounts for eventual multiple diffusion directions in a voxel. Random seed points are chosen within each voxel, and streamlines are propagated in two opposite directions coherently with the local diffusion directions and using a fixed step size. The propagation is constrained within the white matter by means of a *high-resolution binary mask* derived from the previous brain segmentation, and it is halted when a stopping criteria is met: (i) reaching the white-grey matter interface, and/or (ii) incoherence between diffusion directions in neighbouring voxels (curvature constraint). The reconstructed streamlines can be further processed, either by filtering on the basis of their length or by smoothing their trajectories with spline basis functions.

**Figure 7:**
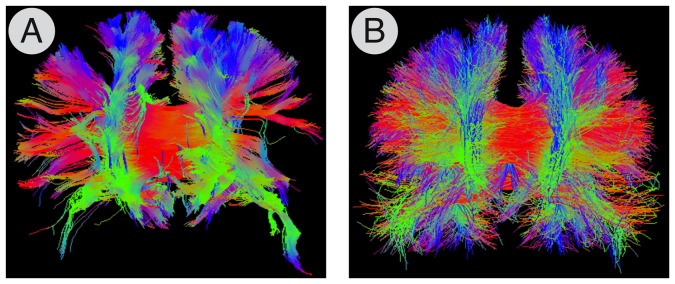
Whole-brain tractography. Example tractograms estimated with (A) the standard *deterministic streamline* and (B) the *global tractography* approach [26].

Streamline-based algorithms are the most used in practice since they are very fast and conceptually simple, and they have been shown to consistently recover the major fibre bundles of the brain [23]. However, this approach is known to be very sensitive to noise and prone to cumulative propagation errors which considerably affect the results. Alternative approaches such as *probabilistic* [24], [25] or *global* [26] *tractography* have been proposed to overcome these limitations. At the time of writing, the aforementioned methods are in the process of being included in the pipeline. Figure 7 presents two example reconstructions performed with two different tractography algorithms.

Concerning the *seeding approach* used for tracking, it is worth noting that each method adopts its own strategy and thus the seeding is very package-specific. For instance, using the default streamline tractography implemented in CMP, 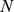 seed points per maxima are randomly chosen in each voxel, while if using MrTrix implementation then 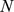 seed points per seed mask are used instead. If probabilistic tractography is adopted, then, tracking is done starting the fibres from a specific ROI and hence 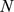 seed points per region are randomly selected. Interestingly, in global tractography [26] no seeding strategy is used in practice, as the fibres are iteratively reconstructed all together with a minimization procedure. However, where possible, CMP offers the possibility to modify the parameters controlling the seeding procedure.

#### Connectome creation

Finally, a connectome, 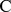, is estimated by combining whole-brain tractography with the cortico-sub-cortical segmentation result. Each reconstructed fibre trajectory which intersects the white-grey matter interface can be assigned to a specific anatomical connection between a pair of regions 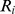 and 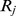. This trajectory will contribute to the cell 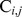 of the connectivity matrix. Since diffusion is a symmetric process, the matrix 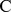 is symmetric, too. To create a connectome CMP uses the NetworkX library (networkx.lanl.gov), which is a powerful Python package for creating and manipulating complex networks.

Although ROI to ROI tracking is not natively supported in CMP at the moment, it is worthy to note that a user interested in studying specific ROI to ROI connectivity can easily filter a posteriori the tracts of interest and manually perform all the desired analyses, as all the necessary information, i.e. whole brain tractography and ROI information, has been computed and is stored in the CMP folder structure.

The number of fibres connecting two regions is a first and simplistic measure of connectivity, but other quantities can be used to characterise more adequately the connectivity strength among every pair of regions. For instance, one can use the average value along the fibre tracts of any diffusion-derived scalar map (e.g. FA, ADC etc) and build a *weighted connectivity matrix* accordingly, as illustrated in figure 6, opening the way to perform *multi-variate* analyses of the brain connectivity. CMP natively offers this possibility by using all the scalar maps previously computed, but additional means for quantifying the connectivity can be easily incorporated.

#### Execution time and accuracy of the estimated connectomes

The Connectome Mapper offers different options to compute connectomes. The choice of which processing steps to execute is dependent on the acquisition modality adopted (DTI, QBI, DSI etc) and represents a trade-off between the power and the sensitivity of the selected methods and the computational burden required. For instance, the *execution time* needed to estimate a connectome is highly dependent on the individual algorithms selected at each stage. As a rule of thumb, a full processing usually requires between 12 and 72 hours of computation per subject on a normal workstation. In fact, the whole-brain segmentation computed with Freesurfer normally takes 12–24 hours and fiber-tracking can require up to 48 hours if probabilistic tractography is performed with default parameters. As reference, deterministic streamline would require 1–3 minutes for the same input data.

The *quantitative comparison* of all these possible approaches (reconstruction, tractography etc) with respect to the estimation of connectomes is still quite an open question in the diffusion MRI community but recent convergent efforts are pointing in this direction [27], [28]. These studies compared the performances of several reconstruction methods and tractography algorithms on a realistic phantom dataset for which the ground-truth was known. However, to our knowledge, no comprehensive studies have been conducted to compare all these methods included in the CMP on real brain data. The final choice of the processing stages is left to the user.

### Export and analysis

Connectomes generated with CMP are internally stored as graph objects in Python pickle format and can be directly analysed using NetworkX and the Matplotlib library (matplotlib.sourceforge.net). NetworkX offers many general purpose algorithms to explore graphs, e.g. Shortest Path and Max Flow, as well as tools to compute local and global network properties, e.g. degree, clustering coefficient etc. In order to favour data sharing and perform more sophisticated analyses with specialised tools, exporters to the most common file formats are available.

By default connectomes are saved in CFF format, which is a file format specifically designed to store and share multi-modal connectome datasets [29]. Data stored in this format can be visualised with the Connectome Viewer (www.connectomeviewer.org), which also offers basic tools to analyse and compare connectomes, such as the Network Based Statistics test [15] and the multi-scale adaptive strategy described in [30]. Connectivity matrices can be exported to Matlab as MAT-files and fed to the Brain Connectivity Toolbox (www.brain-connectivity-toolbox.net), which is a powerful toolbox containing a large selection of network measures for the characterisation of brain connectivity datasets. Finally, CMP can interface also with a lot of general purpose software packages for the analysis of graphs, e.g. Cytoscape (www.cytoscape.org) or Gephi (www.gephi.org), since data can be saved in generic file formats such as GraphML, GML and DOT.

## Results and Discussion

### Applications to clinical studies

The Connectome Mapper was successfully employed in [31] to investigate the structural plasticity of the contra-lesional motor network after an ischemic stroke event. DSI acquisitions and clinical examinations were performed in 12 patients in the acute phase, at 1 and 6 months after the stroke onset. Structural reorganisation of the brain connectivity was assessed analysing the longitudinal time evolution of the motor sub-network and using the GFA as a mean to quantify the strength of the connections. The GFA measured in the acute phase together with age and routine motor scores (National Institute of Health Stroke Score, Functional Independence Measure and modified Rankin Scores) were found to be a strong predictor of the motor outcome at six months after stroke (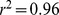, 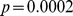). This predictive model of the post-stroke functional recovery was estimated with linear regression (GLM) of the diffusion MRI data and the clinical evaluations collected at the time of the stroke onset. This study represented a “proof of principle” that connectome-like analysis may provide reliable information for personalised rehabilitation planning after an ischemic motor stroke event.

In [30] DTI was used for comparing the structural connectivity in two groups of subjects affected by the 22q11.2 deletion syndrome, distinguished by their IQ scores. Two different approaches were used to quantify the connectivity strength and creating distinct weighted connectivity matrices: (i) using the mean FA along the trajectories connecting a pair of regions and (ii) the so called “connection density” defined as 
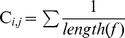
, computed over all the fibre tracts 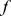 connecting two regions 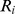 and 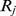. It was shown that there was a specific alteration of the connectivity in the striatal structure (composed of the caudate and the putamen) affecting the cortico-striatonigral-thalamocortical circuit and this alteration might be the cause of the cognitive impairment in the 22q11.2 subjects with the low IQ.

### Strengths and weaknesses

The Connectome Mapper presents some important characteristics from which the neuroimaging community might benefit. The CMP integrates the most popular state-of-the-art processing and analysis tools, while keeping a highly modular structure which makes the integration of additional/custom functionalities possible. Each module can be independently executed with the help of a user-friendly GUI and the output can be visually inspected after each step, allowing the user to always keep an eye on the processed data and quickly identify the source of problems. If something went wrong, the GUI helps the user in tuning the parameters and re-run those steps which eventually produced unsatisfactory results. Moreover, the Connectome Mapper has an active support community of users helping each other in solving issues, exchanging feedbacks and suggesting ideas for improving the software. The forum is available at the address groups.google.com/group/cmtk-users.

As highlighted in figure 1, using CMP the creation of connectomes is completely independent from the acquisition scheme adopted (DTI, DSI etc) and several processing alternatives are available during the workflow. For instance diffusion data can be reconstructed with several techniques, a collection of anatomical atlases can be used and whole brain tractography can be performed choosing from different algorithms. Our software guides the user through all these possible choices and provides a smooth processing regardless of the choices undertaken. From this point of view CMP is remarkably flexible and powerful at the same time, offering a comprehensive framework to perform *multi-variate*, *multi-scale* and *multi-modality* (see below) connectome investigations. The integration of the atlas described in [14] allows to map connectomes at multiple scales. Furthermore, multi-variate connectivity matrices can be created by using different measures for quantifying the connectivity strength of the edges in the brain network (e.g. number of connecting pathways, average FA along them etc).

The Connectome Mapper simplifies the creation of connectomes and makes it a straightforward process even for users not familiar with pipelining languages, for clinician and for researchers working in different domains. At the same time, however, it fulfils the needs of advanced users in charge of analysing huge amount of data, offering them the possibility to save all the parameters in script files and create a batch job to automatically process all the data. A detailed documentation is available at www.cmtk.org/connectomemapper, including a step-by-step guide for installation together with some sample datasets to start testing the pipeline. Each processing step is described in detail and some intermediate results as shown as well. In case of problems, a forum is also available for support. Connectomes can be exported to many file formats, and so CMP is natively compatible with the most popular software packages used in this field. On one side this guarantees the possibility to perform complex network analyses with specialised software packages, and on the other side it might facilitate the sharing of the results between groups in the diffusion community.

Our software pipeline is developed in Python and is released as open-source. The processing steps and the implementation details are then completely transparent to the user and this might facilitate contributions, fixing of bugs and improvements from external developers. However, most of the tools on which it relies (e.g. Fsl and Freesurfer) natively run only on Linux-based systems and, for this reason, CMP is not multi-platform and has been tested so far only on Linux distributions such as Ubuntu for 32bit and 64bit. Some successful attempts to run CMP on different platforms have been reported by some users on the forum. Anyway, as nowadays virtual machine technologies are quite efficient, CMP can be easily run on virtually any system and this does not constitute then a real limitation.

### Availability and future directions

The Connectome Mapper is already available for download and use at www.cmtk.org, and many users already employed it in their studies. The method has been validated in the work of [14] and the code was internally tested applying CMP in many possible scenarios. However, an online forum is available for submitting bugs, comments or feedbacks, for requesting new features, or for simply asking for help. We directly offer support for correcting bugs and for fixing any possible incompatibility which might arise in future due to new releases of the software used in the CMP, e.g. Fsl, Freesurfer etc.

At the time of writing, some modules are still in the beta phase since not fully tested, but are scheduled to be released in short time. It is the case for instance of the reconstruction of the intra-voxel diffusion structure with advanced techniques implemented in Camino and MrTrix, but also of probabilistic and global tractography.

We believe that the Connectome Mapper will represent in the future a solid framework for multi-modal connectome investigations, integrating and merging several imaging modalities, e.g. functional MRI, electroencephalography (EEG), electrocorticography (ECoG) etc, for studying the brain connectivity from different points of view at the same time. CMP already includes a basic module for the pre-processing of fMRI data, typically acquired during resting-state experiments. Initially the fMRI volumes are motion-corrected and averaged. Then, similarly to the registration module described before, the T1-weighted image is registered to the mean fMRI volume and the transformation applied to tissue masks derived from the chosen anatomical atlas. Once the fMRI time points have been realigned, and the cortical parcellation registered to the functional space, an average functional time series can be computed from each cortical region. These average time series are organised into 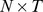 matrices, with 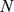 number of cortical and subcortical regions and 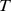 number of time points, and then can be directly fed to the Connectivity Decoding Toolkit (miplab.epfl.ch/richiardi/software.php) developed by [8] to create robust multi-resolution functional connectivity matrices corresponding to different wavelet sub-bands. It is worth noting that in CMP the very same anatomical atlas is used for creating both structural and functional networks, and so the Connectome Mapper might become a very powerful tool for multi-modal brain connectivity analyses.

## Conclusion

In this paper we presented a comprehensive software pipeline specifically designed for easily mapping connectomes from diffusion MRI data using state-of-the-art tools developed in the field. The workflow natively supports the most popular diffusion acquisition schemes (e.g DTI, QBI, DSI) and several processing alternatives are available at each step (e.g. deterministic vs probabilistic tractography). The processing runs smoothly regardless of the methods chosen and a user-friendly GUI helps the user in configuring all the parameters required at each step and visually inspecting all the processed results. The modular structure of our software is highly flexible, and custom or additional algorithms can be incorporated. We believe that our software might play an important role in the field of brain connectivity analyses, for that applied researchers will not have to spend time in developing their own processing pipelines, but they can simply focus all their energies in the results of the analyses.
